# Fungal translocation measured by serum 1,3‐ß‐D‐glucan correlates with severity and outcome of liver cirrhosis—A pilot study

**DOI:** 10.1111/liv.15648

**Published:** 2023-06-19

**Authors:** Matthias Egger, Angela Horvath, Florian Prüller, Peter Fickert, Malcolm Finkelman, Lisa Kriegl, Henning Grønbæk, Holger Jon Møller, Juergen Prattes, Robert Krause, Martin Hoenigl, Vanessa Stadlbauer

**Affiliations:** ^1^ Division of Infectious Diseases, Department of Internal Medicine Medical University of Graz Graz Austria; ^2^ Biotechmed‐Graz Graz Austria; ^3^ Division of Gastroenterology and Hepatology, Department of Internal Medicine Medical University of Graz Graz Austria; ^4^ CBmed Center of Biomarker Research Graz Austria; ^5^ Clinical Institute of Medical and Chemical Laboratory Diagnostics Medical University of Graz Graz Austria; ^6^ Clinical Development, Associates of Cape Cod, Inc Falmouth Massachusetts USA; ^7^ Department of Hepatology Aarhus University Hospital Aarhus Denmark; ^8^ Department of Gastroenterology Aarhus University Hospital Aarhus Denmark; ^9^ Department of Clinical Biochemistry Aarhus University Hospital Aarhus Denmark; ^10^ Clinical and Translational Fungal‐Working Group University of California San Diego San Diego California USA; ^11^ Division of Infectious Diseases and Global Public Health University of California San Diego San Diego California USA

**Keywords:** 1,3‐β‐D‐glucan, gut, liver cirrhosis, microbiome, mycobiome

## Abstract

**Background & Aims:**

On a global scale, liver cirrhosis is attributable to ~1 million deaths per year. This systemic disease comes along with diverse sequelae, including microbiota alterations, increased gut permeability and translocation of microbial components into the systemic circulation. Alongside the extensively studied influence of bacterial translocation and its host–pathogen interactions, far less is known about the role and impact of fungal components once having crossed the intestinal barrier.

**Methods:**

Including 70 patients with different aetiologies of liver cirrhosis, we investigated the relationship between fungal translocation, measured by 1,3‐β‐D‐glucan (BDG), and biomarkers of gut integrity, inflammation and severity/outcome of liver disease.

**Results:**

Patients with cirrhosis Child–Pugh class (CPC)‐B were more likely to have positive serum BDG (aOR 5.4, 95% CI 1.2–25.2) compared to patients with cirrhosis CPC‐A. BDG showed a moderate positive correlation with several markers of inflammation (sCD206, sCD163, Interleukin 8, interferon‐gamma‐induced protein). Mortality differed significantly between patients with positive versus negative BDG (log‐rank test, *p* = 0.015). The multivariable Cox regression model yielded an aHR of 6.8 (95% CI 1.8–26.3).

**Discussion:**

We observed trends for increased fungal translocation depending on the severity of liver cirrhosis, an association of BDG with an inflammatory environment and the adverse effects of BDG on disease outcome. In order to gain more in‐depth knowledge about (fungal‐)dysbiosis and its detrimental consequences in the setting of liver cirrhosis, these trends need to be studied in more detail including prospective sequential testing in larger cohorts together with mycobiome analyses. This will further elucidate complex host–pathogen interactions and potentially introduce points of application for therapeutic interventions.

**Abbreviations**
AUC: area under the curve
BDG: 1,3‐β‐D‐Glucan
CI: confidence interval
CPC: Child–Pugh class
CTP: Child–Turcotte–Pugh
ELISA: enzyme‐linked immunosorbent assay
IFN: interferon
IL: interleukins
IP: interferon‐gamma induced protein
IQR: interquartile range
LBP: lipopolysaccharide‐binding protein
MELD‐Na: model of end‐stage liver disease natrium
MIG: monokine induced by gamma‐interferon
ROC: receiver operating characteristic
TNF: tumour necrosis factor

**Key points**

Liver cirrhosis is a severe systemic disease leading to alterations in the gut microbiome composition and increased translocation of microbial components into the systemic circulation. These components may trigger inflammatory responses resulting in worsened clinical disease course and outcome. The presence of the fungal cell wall component 1,3‐ß‐D‐glucan in serum was higher in patients with more advanced stages of cirrhosis, associated with markers of (hepatic) inflammation, and was a positive predictive factor for mortality.

## 1 INTRODUCTION

Liver cirrhosis is a severe systemic disease widely prevalent in low‐, middle‐, and high‐income countries and attributable to ~1 million worldwide deaths annually.^1^ Despite major efforts in cirrhosis‐related research having led to an improved understanding of pathogenesis, diagnosis and treatment, various aspects of disease‐related complications remain to be understood in detail. Of particular interest are the relationship between the gut microbiota and microbial translocation acting as a potential driver of persistent inflammation involving adverse effects on clinical disease course and outcome.

Fungi are commensals of the human skin, respiratory tract and gut. The fungal part of the human gut microbiota is referred to as the mycobiota. Alongside bacteria, fungi account for the second most abundant population of the gut microbiota.^2^ Predominant human intestine fungal species comprise ascomycetes like *Candida albicans* and other yeasts as well as moulds like *Aspergillus* spp. and *Fusarium* spp.^3^ One common characteristic feature shared by the above‐mentioned fungi is the production of the polysaccharide 1,3‐ß‐D‐glucan (BDG), a fungal cell wall component. To date, BDG is mainly serving as a pan‐fungal serum biomarker for the diagnosis of invasive infection, however, the presence of BDG in blood has also been correlated with gut dysbiosis, immune activation, persistent inflammation, microbial translocation and prediction of non‐AIDS events in patients with HIV infection.^4^, ^5^, ^6^, ^7^, ^8^, ^9^ While research in liver cirrhosis has mainly focused on bacterial–host interactions, the fungal part and potential cross‐kingdom microbial interactions remain enigmatic. It has been acknowledged that liver cirrhosis and its multifaceted sequelae, including portal hypertension leading to intestinal hypoperfusion and gut microbiome dysbiosis, are associated with increased gut permeability.^10^ Alterations of the gut barrier result in increased translocation of bacterial as well as fungal components, which is associated with an increased risk of infections.^10^, ^11^, ^12^, ^13^ Further, the presence of microbial elements in the bloodstream induced a hyperinflammatory state and an increased rate of complications in cirrhosis patients.^14^ Narrowing down on the fungal part, fungal translocation measured by serum BDG levels had various effects on immunologic pathways as well as the microbiome composition of mice chronically exposed to ethanol administration, and a small cohort of patients with alcoholic cirrhosis.^15^ Findings included (a) chronic alcohol administration led to intestinal fungal overgrowth and increased translocation of BDG into the systemic circulation, (b) BDG‐induced liver inflammation and expression of interleukins leading to hepatocyte damage and aggravation of ethanol‐induced liver disease in mice, (c) levels of extraintestinal exposure of fungal components in humans was accompanied by increased mortality and (d) local antifungal treatment in mice had positive effects on points a–c.^15^

Larger scale evaluations that correlate fungal translocation with the severity of liver cirrhosis, extent of the “leaky gut”, and disease outcome in humans are lacking. For this aim, we prospectively measured levels of serum BDG and investigated associations with additional biomarkers for gut integrity, microbial translocation, inflammation and severity/outcome of patients with liver cirrhosis.

## 2 METHODS

### 2.1 Study design

Biosamples were collected as part of the study “Influence of Probiotics on Infections in Cirrhosis” between July 2012 and March 2014. For this study, samples from the last timepoint (after 6 months of washout of the study medication) were used. Of the initial cohort (*n* = 101) we could only include 70 patients solely due to the fact of restricted sample availability. All samples were transported to the lab on ice and immediately aliquoted and frozen at −80°C until analysis. Between March and July 2022, 70 serum samples from 70 different patients with underlying liver cirrhosis were prospectively measured for BDG levels at the Medical University of Graz. Child–Turcotte–Pugh score as well as the model of end‐stage liver disease natrium (MELD‐Na) was used to determine the severity of cirrhosis. The study was approved by the research ethics committee of the Medical University of Graz (23‐096 ex 10/11) and registered at clinicaltrials.gov (NCT01607528). All procedures were performed according to the Declaration of Helsinki.

### 2.2 Measurements

BDG was tested according to previously described methods using reagents from the Fungitell® Assay (Associates of Cape Cod, Falmouth, MA).^16^ Commercially available enzyme‐linked immunosorbent assays (ELISA) were used according to manufacturers' protocols for measuring sCD14 in lithium‐heparinized plasma (R&D Systems), zonulin in stool and serum, calprotectin in stool (Immundiagonstik), diaminooxidase in serum (Immundiagnostik) and lipopolysaccharide‐binding protein in EDTA plasma [LBP (Hycult Biotech)]. Levels of sCD163 in plasma samples were measured by an in‐house sandwich enzyme‐linked immunosorbent assay (ELISA) using a BEP‐2000 ELISA analyser (Dade Behring) as previously described.^17^, ^18^ Levels of sCD206 in plasma samples were measured by an in‐house ELISA assay as previously described.^19^ For endotoxin measurement, HEK‐Blue LPS Detection Kit 2 (Invivogen, Toulouse, France) was used as previously described.^20^ The differential sugar absorption test was performed with lactulose (10 g) and mannitol (5 g) and analysed by nuclear magnetic resonance spectroscopy as previously described.^20^ Interleukins (IL) 1β, 6, 8, 10, tumour necrosis factor (TNF) α, interferons (IFN) α, β, γ, interferon‐gamma induced protein (IP) and monokine induced by gamma‐interferon (MIG) were assessed in sodium citrate plasma using ProcartaPlex® Multiplex Immunoassay (Affimetrix, Vienna, Austria) according to manufacturer's instruction and measured with a Bio‐Plex 200 (Biorad, Hercules, USA). For BDG positivity, we used the cut‐off of ≥80 pg/mL, which is recommended by the manufacturer for diagnosing invasive fungal infections. Importantly, this is not the detection limit for the assay, which is 15.4 pg/mL^16^; the test is linear between about 15 pg/mL and about 250 pg/mL, with modest deviations from linearity below 15 pg/mL (higher slope) and above about 300 pg/mL (slightly lower slope). To date, definition of an appropriate cut‐off for fungal translocation is lacking.^4^ Thus, relying upon a high cut‐off value, such as 80 pg/mL, provides a more conservative estimate of the utility of the BDG titre where a significant relationship with outcome, symptom severity or another biomarker was also observed in various other non‐fungal infection disease states [e.g., CKD, inflammatory bowel disease, COPD, Lupus^21^, ^22^, ^23^, ^24^].

### 2.3 Statistical analysis

Continuous variables are reported as median plus interquartile range (IQR). Correlation between various biomarkers was calculated using Spearman's rho correlation analysis due to the non‐normal distributions. Correlation matrixes were calculated and graphically depicted as heat diagrams. Logistic regression was used to assess predictors of positive 1,3 BDG serum levels (≥80 pg/mL). Univariate analysis of relevant parameters for differentiating Child–Pugh class (CPC) A versus CPC‐B was performed. For multivariable analysis, we used backward model selection to adjust for potential confounders and considered parameters significant at *p* ≤ 0.2. Receiver operating characteristic (ROC) curve analyses were performed and area under the curve (AUC) values were calculated including 95% confidence intervals (CI) for the outcomes CPC‐A versus CPC‐B and overall mortality versus survival. Overall survival was calculated with a Kaplan–Meier estimator. For the comparison of survivor functions between the two study groups, we used the log‐rank test. To investigate the association of risk factors with survival, univariable and multivariable Cox models were estimated. For multivariable analysis, we used backward model selection to adjust for potential confounders and considered parameters significant at *p* ≤ 0.2. The proportionality of the hazard assumption was evaluated by fitting an interaction between a variable of interest and linear follow‐up time. The proportional hazards assumption was tested using the Schoenfeld residuals test for the overall model and individual covariates. The resultant model and all other Cox models did not significantly violate the proportional hazards assumption for individual covariates or the global model. A two‐sided *p*‐value of <0.05 was taken as the cut‐off for statistical significance. All statistical analyses were performed using R software, version 3.3 (R Development Core Team) and GraphPad Prism 9 Version 9.4.1.

## 3 RESULTS

### 3.1 Baseline characteristics

A total of 70 outpatients with underlying liver cirrhosis were included. None of the patients had clinical signs or symptoms of candidemia, invasive candidiasis or oral/oesophageal candidiasis. Aetiologies of cirrhosis were alcoholic in 36 patients, chronic hepatitis C virus infection in 12 patients and others in 22 patients (Table 1). The severity of cirrhosis determined by Child–Turcotte–Pugh (CTP) score matched 49 patients with cirrhosis CPC‐A and 21 patients with cirrhosis CPC‐B. Descriptive statistics of measured biomarkers and inflammatory parameters are shown in Tables 1 and S1.

**TABLE 1:** Characteristics of the study cohort and measurements of laboratory values.

	Study cohort (*n* = 70)
	*N* (%) or median (IQR)
Age, years	58 (51–64)
Sex—no. (%)	
Male	50 (71)
BMI, kg/m^2^	26.4 (24.7–30.8)
Cirrhosis aetiology (%)	
Alcoholic	36 (51)
Chronic HCV infection	12 (17)
Others^a^	22 (31)
Child–Pugh Class A	49 (70)
Child–Pugh Class B	21 (30)
Model of end‐stage liver disease score (SD)	11.1 (3.8)
Charlson Comorbidity Index (SD)	1.5 (1.9)
Mortality at end of the follow‐up (*n*)^c^	
Dead^b^	11 (16)
Alive	54 (77)
Laboratory measurement in serum	
BDG pg/mL (<60 pg/mL)	15 (15–65)
Zonulin ng/mL	40 (25–49)
sCD14 μg/L	1.9 (1.6–2.3)
Diaminooxidase U/mL	15 (13–20)
Lactulose‐Mannitol‐Ratio^d^	0.1 (0.45)
LBP ng/L	18 (15–22)
LPS EU/mL	0.8 (0–8.7)
Bilirubin mg/dL	1.2 (0.8–2.2)
Albumin g/dL	4.1 (3.5–4.5)
Creatinine mg/dL	0.8 (0.7–1.0)
Natrium mmol/L	139 (137–141)
INR	1.2 (1.1–1.4)
CRP mg/L	2.8 (0.9–4.7)
Ferritin μg/L	105 (53–219)
Thrombocytes 10^9^/L	106 (70–160)
Neutrophils 10^9^/L	60 (54–65)
Stool	
Calprotectin μg/g	146 (36–332)
Zonulin ng/mL	75 (60–95)

Abbreviations: BDG, 1,3‐Beta‐D‐Glucan; CRP, C‐reactive protein; HCV, hepatitis C virus; LBP, lipoprotein‐binding protein; LPS, lipopolysaccharide.

^a^ (*n*) Hepatitis B virus (4), hemochromatosis (3), primary biliary cholangitis (3), Morbus Wilson (3), non‐alcoholic steatohepatitis (3), alpha‐1 antitrypsin deficiency (1), unknown aetiology (5).

^b^ Causes of death: decompensated cirrhosis (6), sepsis (2), hepatocellular carcinoma (1), cholangiocellular carcinoma (1), unknown cause of death (1).

^c^ Missing data in five patients; mortality for observational period; 90‐day mortality 4% (3/70), 28‐day mortality 0% (0/70).

^d^ Mean (standard deviation).

### 3.2 Relationship between 1,3‐β‐D‐glucan levels with CP and MELD score

**FIGURE 1:**
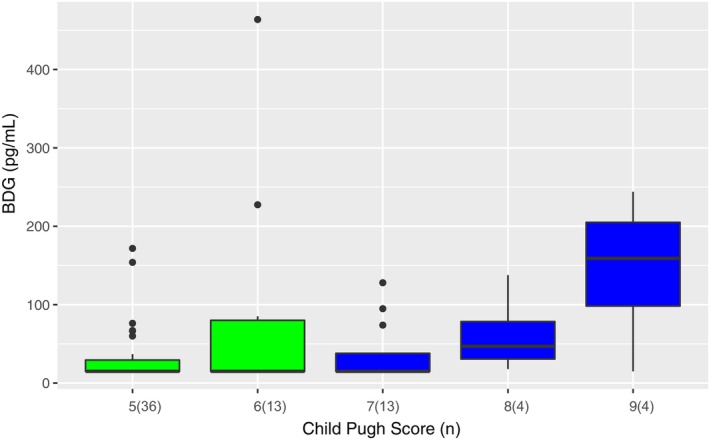
Boxplots matched with Child–Pugh score. Green Box Whisker plots indicate Child–Pugh class A; Blue Box Whisker plots indicate Child–Pugh class B.

Serum BDG levels were lower (median = 15 pg/mL; IQR = 15–37) in those with CPC‐A versus those with CPC‐B (median 26 pg/mL; IQR = 15–126; *p* = 0.042, Mann–Whitney U) and BDG levels increased with an increase in CTP score (Figure 1).

In univariate analysis, patients with cirrhosis CPC‐B had 3.6‐fold higher odds (95% CI 1.03–12.3, *p* = 0.04) for a positive BDG (i.e., >80 pg/mL) result compared to CPC‐A. We considered markers of (chronic) inflammation (C‐reactive Protein, neutrophil count, thrombocytes, calprotectin in stool, ferritin) as potential confounders of the primary predictor to evaluate whether CPC and not a general inflammatory state are the actual drivers of BDG elevation (other permeability biomarkers not considered due to lack of correlation). Using backward model selection ferritin, thrombocytes and absolute neutrophil count, remained in the final model. In the adjusted model, OR for the positivity of BDG was 5.4 (95% CI 1.2–25.2) when comparing CPC‐B to CPC‐A.

For differentiating CPC‐B versus CPC‐A, ROC curve analysis yielded an AUC of 0.635 (95% CI 0.484–0.785) for BDG, resulting in a slightly higher AUC value compared to other biomarkers (Figure S1A).

### 3.3 Relationship among biomarkers, inflammatory markers and laboratory values with 1,3‐β‐D‐glucan

**FIGURE 2:**
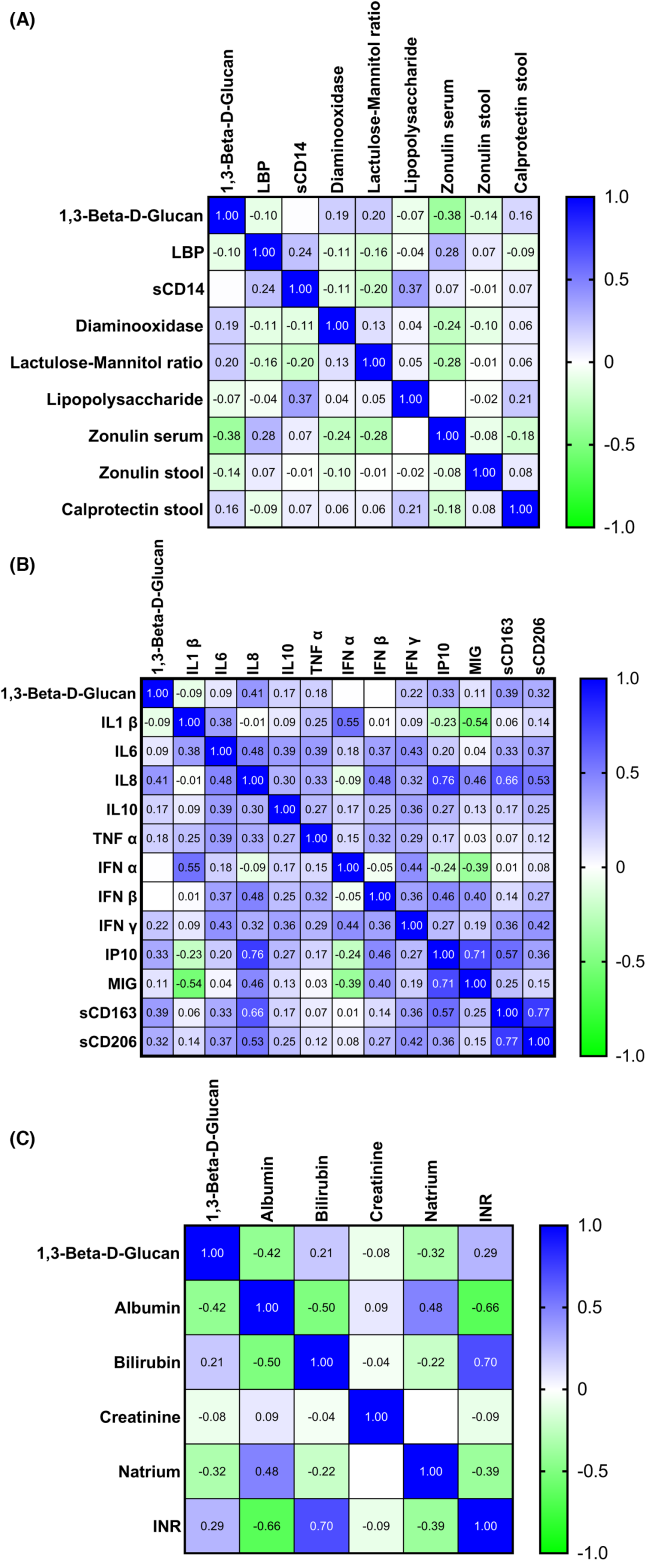
Spearman correlation matrix. (A) 1,3‐β‐D‐glucan and various gut permeability markers, (B) 1,3‐β‐D‐glucan and various interleukins and (C) 1,3‐β‐D‐glucan and laboratory values of Child–Turcotte–Pugh and MELD–Na score. IFN, interferon; IL, interleukin; INR, international normalized ratio; IP, interferon‐gamma induced protein; LBP, lipopolysaccharide‐binding protein; MIG, monokine induced by gamma‐interferon; TNF, tumour necrosis factor.

Zonulin in serum showed a moderate negative correlation (*ρ* −0.38, *p* = 0.001) with BDG, while no significant findings were obtained for other permeability or translocation markers studied (sCD14, diaminooxidase, lactulose‐mannitol ratio, LPS, LBP, zonulin or calprotectin in stool; Figure 2A).

Due to the inflammatory properties of BDG when entering the bloodstream, we investigated correlations between various inflammatory markers, interleukins and BDG. We found significant results for sCD163 (*ρ* 0.39, *p* < 0.001), sCD206 (*ρ* 0.32, *p* = 0.007), IL 8 (*ρ* 0.41, *p* < 0.001) and IP10 (*ρ* 0.33, *p* = 0.004), while IL 1β, IL 6, IL 10, TNF α, IFN α, IFN β, IFN γ and MIG yielded no significance (Figure 2B).

Examining the correlation of BDG with single laboratory values of CTP and MELD‐Na score, we observed significant correlations between BDG with albumin (*ρ* −0.42, *p* < 0.001), INR (*ρ* 0.29, *p* = 0.02) and natrium (*ρ* −0.32, *p* = 0.007), while creatinine and bilirubin yielded no significant association (Figure 2C).

### 3.4 Prediction of survival

Eleven patients died after a median of 588 days following enrollment (IQR 90–934 days; observation period in survivors median 797 days, range 229–1128 days). ROC curve analysis for the prediction of overall mortality vs. survival showed an AUC of 0.622 (95% CI 0.420–0.820) for BDG (Figure S1B). Other biomarkers, namely LPS (AUC = 0.758 95% CI 0.602–0.915), sCD14 (AUC = 0.691 95% CI 0.497–0.884) and calprotectin (AUC 0.692 95% CI 0.543–0.842) yielded higher AUC values for the prediction of death.

**FIGURE 3:**
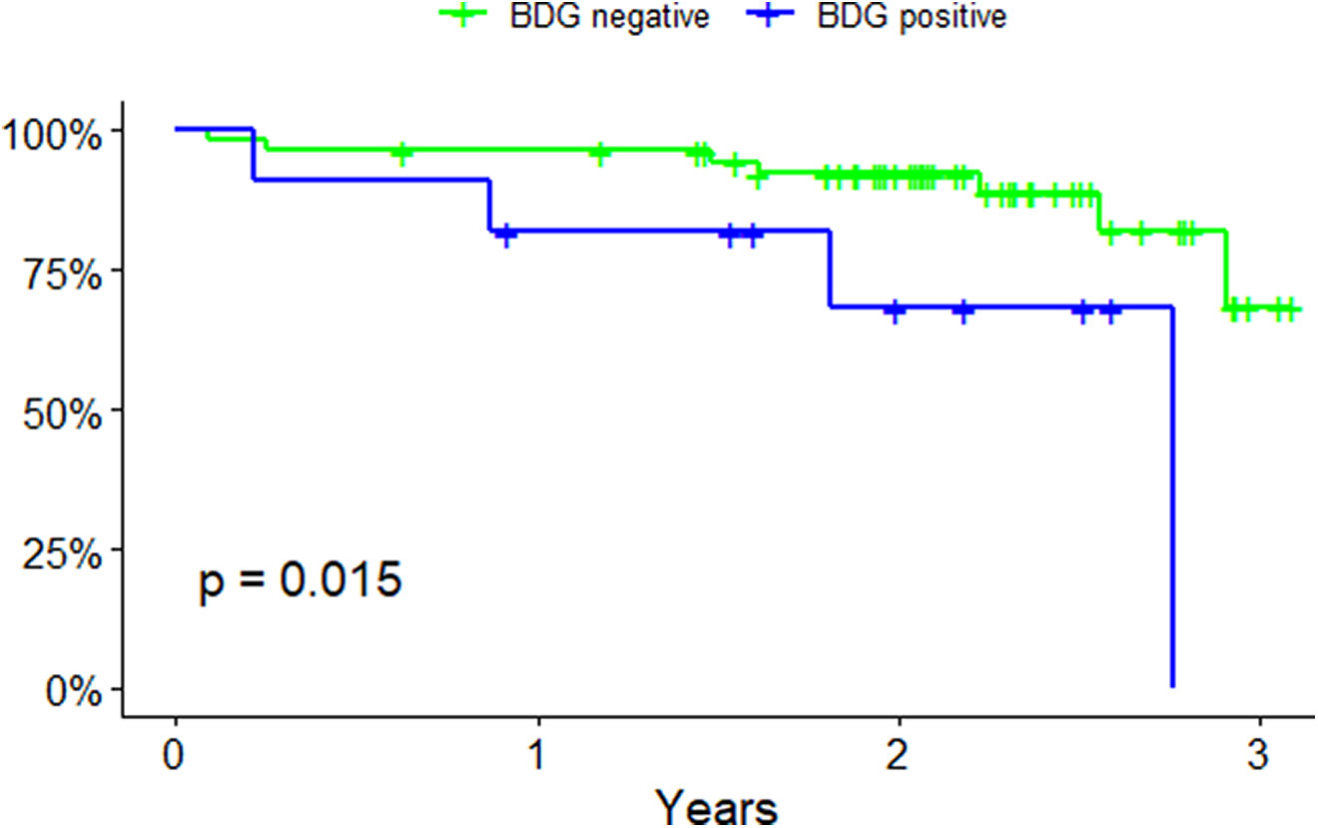
Kaplan–Maier survival estimation.

Using Kaplan–Meier estimator for survival, we found a significant difference between survival in patients with positive BDG compared to patients with negative BDG (log‐rank test *p* = 0.015) (Figure 3). In a univariable Cox regression model, BDG positivity showed a hazard ratio of 4.3 (95% 1.2–15.2) for mortality. After adjusting for potential confounders (age, sex, MELD score, CTP score, Charlson Comorbidity index, Ferritin, Thrombocytes and Neutrophils) and using backward model selection (*p* < 0.2), MELD‐Na score and Ferritin remained in the final model. The adjusted hazard ratio for death in patients with positive BDG was 6.8 (95% CI 1.8–26.3).

## 4 DISCUSSION

We found an association between serum BDG levels with liver disease severity and mortality in patients with liver cirrhosis. Patients with positive BDG had 5.4 times higher odds of having more advanced disease (CPC‐B compared to CPC‐A). Representation of patients with CPC‐A and CPC‐B only allows the assumption that these associations would be more distinct when compared to patients with CPC‐C. This was observed in a previous pilot study evaluating the BDG levels over time in patients without invasive fungal infections, where constantly higher levels of BDG were observed in a patient with CPC‐C versus another patient with CPC‐B.^13^

The findings of our study are in line with several previous studies that showed the disruptive effects of cirrhosis on gut integrity, alterations in microbiome, intestinal overgrowth of fungal species, and hence translocation of fungal components into the systemic circulation.^12^, ^25^ While fungal translocation may only lead to slight elevations of BDG, with levels often much lower than those found in patients with invasive fungal infections (e.g., median 65 pg/mL, IQR <15–138 pg/mL in ICU patients with suspected *Candida* infections^26^), the biomarker may still be able to predict disease outcomes.^7^, ^27^, ^28^ In an animal cirrhosis model, Yang et al. found higher levels of extraintestinal BDG which led to increased expressions of interleukins and hence inflammation, resulting in hepatocyte damage and aggravation of liver disease.^15^ In our pilot study in a human cohort, serum BDG levels increased with an increase in the severity of cirrhosis, which may indirectly represent gut barrier disruptions and hence a higher amount of BDG crossing the intestinal barrier. BDG was significantly correlated with several markers of (hepatic) inflammation (IL 8, IP10, sCD163 and sCD206) being consistent with the hyperinflammatory environment coming along with cirrhosis in general, but which might be further triggered by microbial, including fungal, translocation. In a cohort with chronic hepatitis C virus infection, BDG levels and sCD163 were also shown to be higher compared to a healthy cohort.^29^ Lastly, we found a significant difference in mortality between patients with positive versus negative BDG. In the multivariable Cox model, we found an aHR of 6.8 (95% CI 1.8–26.3) for death in patients with positive BDG ≥80 pg/mL.

Recent research indicates that serum BDG may serve as a reliable and stable biomarker measuring fungal translocation. As levels of other permeability markers like LPS were shown to vary depending on daytime and food intake,^30^, ^31^ the same was assumed for BDG. Plant‐based BDG is a component of foods such as cereals, mushrooms and yeast and is often utilized as a nutritional supplement. Another pilot study showed that BDG blood levels were not influenced by plant‐origin BDG‐rich nutrition in people with advanced liver cirrhosis, and HIV, indicating that the interpretation of serum BDG levels may not need to be adjusted for nutrition and daytime.^13^ While animal and human studies have demonstrated the role of hepatic clearance in the elimination of circulating BDG^32^, ^33^ and also that a minor component of circulating BDG is cleared renally,^34^ indicating that less secretion or less metabolism may contribute to sustained elevated BDG levels. It has to be emphasized, however, that BDG has to enter the bloodstream via translocation in the first place before the above‐mentioned affects might come into play.

BDG has been studied as a marker of fungal translocation in various clinical settings (e.g., sepsis, haemodialysis, abdominal surgery).^26^, ^28^, ^35^ All these conditions lead to increased gut permeability through different pathophysiological pathways, resulting in microbial translocation, including fungal translocation that can be measured by serum BDG. Besides, detailed investigations between non‐AIDS events and serum BDG levels were conducted and found that elevated serum BDG levels were able to predict and correlated with higher clinical event rates.^7^, ^8^, ^9^

Interestingly, we could not find clear relations between serum BDG and other biomarkers of increased gut permeability. Similar findings were obtained in a very recent study where BDG levels in HIV patients decreased after 1 year of anti‐retroviral therapy.^36^ The absence of correlation with other permeability markers might also be partially explained by another mechanism acting a part, which is a decrease in BDG clearance by the hepatic reticuloendothelial system with increasing hepatic inflammation and hence higher levels in peripheral blood.^32^

In a mouse model, Yang et al. observed that a decrease in intestinal fungal overgrowth by non‐absorbable Amphotericin B did not alter the gut barrier function and permeability remained increased.^15^ These findings may indicate that fungal dysbiosis might not be alone driving the disruption of gut integrity and that other factors such as hypoperfusion of the gut occurring in patients with liver cirrhosis, as well as persistent low‐level inflammation caused by microbial products and BDG entering the bloodstream, and underlying diseases might play important roles as well. Nevertheless, non‐absorbable Amphotericin B led to a decrease in serum BDG levels and had a positive effect on disease severity.^15^

Limitations of our study are the retrospective cohort and the small number of patients. Further, only patients with cirrhosis CPC‐A and B were represented, with low short‐term mortality rates, only allowing interpretation for this constrained extent of cirrhosis. Future studies should evaluate the kinetics of BDG over time in cohorts with CPC‐C cirrhosis and higher mortality rates. The cut‐off for BDG positivity (≥80 pg/mL) was chosen based on the cut‐off for invasive fungal infections. To date, there is no established cut‐off for indicating fungal translocation, hence interpretation is limited. Another limitation of our study is the fact that there is no consensus on the optimal choice of gut permeability markers in cirrhosis. We chose a panel of four permeability markers and three translocation markers which were commonly used in cirrhosis by us and others in the past to overcome this limitation. While we have tested a large set of biomarkers for permeability and inflammation, we did not measure other established markers, such as I‐FABP and REG3alpha, which should be added in future studies. Finally, fungal translocation may differ between different aetiologies of cirrhosis due to varying effects on the components of the intestinal barrier (i.e., mucus, microbes, epithelium, immune cells). For example, ethanol and its toxic properties per se negatively affect the intestinal barrier (e.g., disruption of epithelial tight junctions, dysbiosis of gut microbiota, altered stem cell functions), which might depict a distinct factor in fungal translocation occurring in alcoholic cirrhosis. While in our study BDG positivity was equally distributed between alcoholic cirrhosis and other etiologies, future studies are needed to investigate this in more detail.

In conclusion, we showed that BDG levels were higher in patients with CPC‐B cirrhosis compared to CPC‐A cirrhosis patients, and that higher BDG leveles were associated with mortality. Further, BDG levels significantly correlated with markers of inflammation. In order to gain more in‐depth knowledge about (fungal‐)dysbiosis and its detrimental consequences in the setting of liver cirrhosis, these trends need to be studied in more detail including prospective sequential testing in larger cohorts and micro‐mycobiome studies. This will further elucidate complex host‐pathogen interactions and potentially introduce points of application for timely therapeutic interventions.

## FUNDING INFORMATION

BDG test kits were provided by the Associates of Cape Cod. The clinical cohort was built up as part of the Austrian Science Fund project P 24362.

## CONFLICT OF INTEREST STATEMENT

Malcolm Finkelman was an employee of Associates of Cape Cod, Inc. the manufacturer of the Fungitell® kit. Other authors declare no conflict of interest for this study.

## ETHICS APPROVAL STATEMENT

The study was approved by the research ethics committee of the Medical University of Graz (23‐096 ex 10/11).

## CLINICAL TRIAL REGISTRATION

This trial was registered at clinicaltrials.gov (NCT01607528).

## Supporting information


**Supporting information S1.** Supplementary material

## Data Availability

Data that support the findings of this study are available from the corresponding author upon reasonable request.
