# Supplementary material for: Fungal translocation measured by serum 1,3‐ß‐D‐glucan correlates with severity and outcome of liver cirrhosis—A pilot study
Source: Liver Int. 2023 Jun 19;43(9):1975–83. doi: 10.1111/liv.15648 (PMC10947104; doi:10.1111/liv.15648)

**Online supplemental material**

**Titel :** **Fungal translocation measured by serum** **1,3-ß-D-glucan correlates with severity and outcome of liver cirrhosis – a pilot study**

Matthias Egger^1^, Angela Horvath ^2,3^, Florian Prüller^4^, Peter Fickert^2^, Malcolm Finkelman^5^, Lisa Kriegl^1^, Henning Grønbæk^6^, Holger Jon Møller^7^, Juergen Prattes^1^, Robert Krause^1,10^, Martin Hoenigl^1,,8,9┼^, Vanessa Stadlbauer^2,3┼^

^1^Division of Infectious Diseases, Department of Internal Medicine, Medical University of Graz, Graz, Austria

^2^Division of Gastroenterology and Hepatology, Department of Internal Medicine, Medical University of Graz, Graz, Austria

^3^CBmed Center of Biomarker Research, Graz, Austria

^4^Clinical Institute of Medical and Chemical Laboratory Diagnostics, Medical University of Graz, Graz, Austria

^5^Clinical Development, Associates of Cape Cod, Inc, Falmouth, MA, USA

^6^Departments of Hepatology and Gastroenterology, Aarhus University Hospital, Denmark

^7^Depratment of Clinical Biochemistry, Aarhus University Hospital, Denmark

^8^Clinical and Translational Fungal-Working Group, University of California San Diego, San Diego, CA, United States

^9^Division of Infectious Diseases and Global Public Health, University of California San Diego, San Diego, CA, United States

^10^Biotechmed-Graz, Graz, Austria

^┼^Shared senior authorship

Corresponding author:

Martin Hoenigl, MD, Assoc. Prof.

Division of Infectious Diseases, Department of Internal Medicine,

Medical University of Graz,

Auenbruggerplatz 15, 8036-Graz, Austria

Email: hoeniglmartin@gmail.com

Phone: +4331638531425

**Supplemental Table 1.** Measurements of laboratory values.

|  | **Study Cohort (n=70) median (IQR)** |
| --- | --- |
| IL-1β | 0 (0-0.45) |
| IL- 6 | 0 (0-6.2) |
| IL-8 | 76.9 (18.8 – 154.6) |
| IL-10 | 0.7 (0.4- 0.7) |
| TNFα | 0 (0- 0.42) |
| IFNα | 0.01 (0-0.09) |
| IFNβ | 8.8 (0 – 36.8) |
| IFNγ | 1.1 (0.8 – 1.7) |
| IP10 | 20.2 (11.4 – 61.9) |
| MIG | 65.6 (33.0 – 157.2) |
| sCD163 | 4.9 (3.2 – 6.8) |
| sCD206 | 0.35 (0.26 – 0.48) |

**Supplemental Figure 1.** (a) ROC Curve analysis for the prediction of Child Pugh grade and (b) for the prediction of death in various permeability markers.
(a) (b)


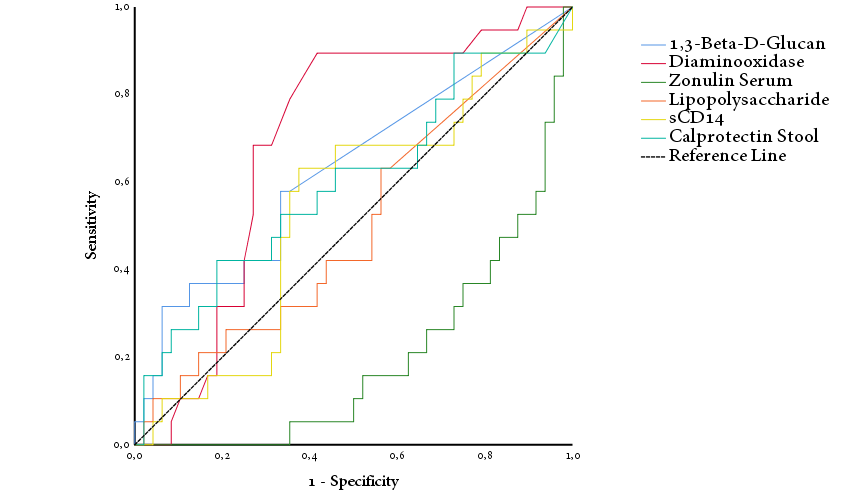

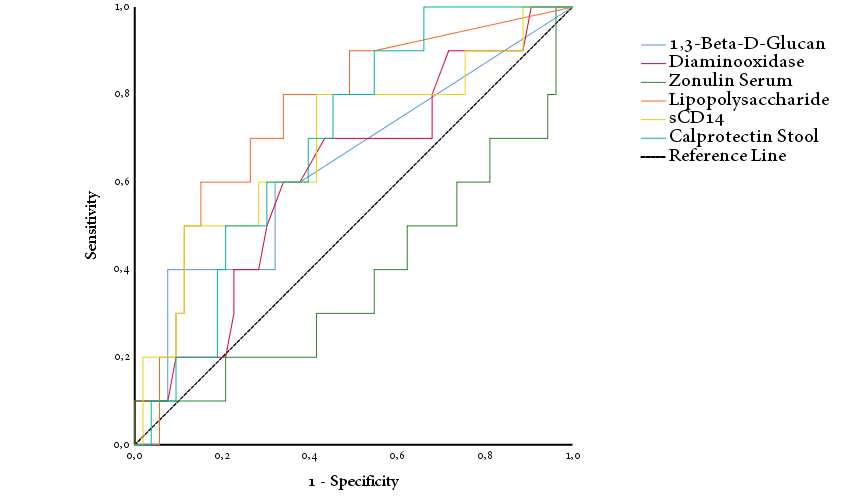

Supplement: Supplementary file 1 — Supporting information S1. Supplementary material [file LIV-43-1975-s001.docx]
